# Hypoxia‐responsive miR‐141–3p is involved in the progression of breast cancer via mediating the HMGB1/HIF‐1α signaling pathway

**DOI:** 10.1002/jgm.3230

**Published:** 2020-06-11

**Authors:** Shanping Sun, Jinglin Ma, Panpan Xie, Zhen Wu, Xingsong Tian

**Affiliations:** ^1^ Cheeloo College of Medicine Shandong University Jinan Shandong Province China; ^2^ Department of Breast and Thyroid Surgery Liaocheng People's Hospital Liaocheng Shandong Province China; ^3^ Department of Breast and Thyroid Surgery, Shandong Provincial Hospital Shandong University Jinan Shandong Province China

**Keywords:** breast cancer, HIF‐1α, HMGB1, hypoxia, miR‐141–3p

## Abstract

**Background:**

Hypoxia‐responsive miRs have been frequently reported in the growth of various malignant tumors. The present study aimed to investigate whether hypoxia‐responsive miR‐141–3p was implicated in the pathogenesis of breast cancer via mediating the high‐mobility group box protein 1 (HMGB1)/hypoxia‐inducible factor (HIF)‐1α signaling pathway.

**Materials and methods:**

miRs expression profiling was filtrated by miR microarray assays. Gene and protein expression levels, respectively, were examined by a quantitative reverse transcriptase‐polymerase chaion reaction and western blotting. Cell migration and invasion were analyzed using a transwell assay. Cell growth was determined using nude‐mouse transplanted tumor experiments.

**Results:**

miR‐141–3p was observed as a hypoxia‐responsive miR in breast cancer. miR‐141–3p was down‐regulated in breast cancer specimens and could serve as an independent prognostic factor for predicting overall survival in breast cancer patients. In addition, the overexpression of miR‐141–3p could inhibit hypoxia‐induced cell migration and impede human breast cancer MDA‐MB‐231 cell growth *in vivo.* Mechanistically, the hypoxia‐related HMGB1/HIF‐1α signaling pathway might be a possible target of miR‐141–3p with respect to preventing the development of breast cancer.

**Conclusions:**

Our finding provides a new mechanism by which miR‐141–3p could prevent hypoxia‐induced breast tumorigenesis via post‐transcriptional repression of the HMGB1/HIF‐1α signaling pathway.

## 1 INTRODUCTION

Breast cancer (BC) is the most commonly diagnosed cancer both in China and worldwide among females, contributing to approximately 15.1% or 24.2% of cancer incidence in China or worldwide, respectively.^1^, ^2^, ^3^ Understanding the basic molecular mechanisms of the initiation and development of BC will be advantageous for optimizing clinical management strategies.

As a low oxygen condition, hypoxia may occur in both normal physiological processes and pathological conditions.^4^ Hypoxia has frequently been reported as a stressor in the tumor microenvironment to confer chemotherapeutic resistance and associate with the poor prognosis of cancer patients.^5^ In conditions of hypoxia, numerous pathways and transcription factors are deregulated, which play a crucial role in aggressive tumor phenotypes, such as angiogenesis, migration, invasion and metastasis.^6^, ^7^ Comprising the most crucial transcription factor, hypoxia‐inducible factor (HIF)‐1, which consists of an inducible α subunit and a constitutively expressed β subunit, is activated by oxygen deprivation.^4^, ^8^ In normal oxygen conditions, the α‐subunit is hydroxylated at proline residues, resulting in its ubiquitination and proteasomal degradation, whereas it evades degradation and assembles with the β‐subunit in conditions of hypoxia, aggregating a heterodimeric transcription factor that can modulate downstream gene expression by binding to target genes with their 5'‐TACGTG‐3' recognition sequence in the nucleus.^4^ Previous investigations corroborate that HIF‐1α can be regulated by high‐mobility group box protein 1 (HMGB1), a non‐histone nuclear protein implicated in various pathologic processes, including angiogenesis, rheumatoid arthritis and carcinogenesis.^9^, ^10^

Some previous studies report that hypoxic microenvironment can disturb the expression of microRNAs, which represent a class of small non‐coding RNAs and function as post‐transcriptional regulators by binding to the 3'‐untranslated region (3'‐UTR) of target genes to weaken the protein translation,^11^ changing the behavior of the tumor cells, including glycolysis, radioresistance, autophagy and epithelial to mesenchymal transition.^12^, ^13^, ^14^, ^15^ In BC, hypoxia triggers miR‐153 to manipulate angiogenesis via modulating the HIF1α/vascular endothelial growth factor axis.^16^ Hypoxia induces the up‐regulation of miR‐210 to increase BC stem cell metastasis and proliferation by targeting E‐cadherin.^17^

In the present study, we aimed to investigate whether the hypoxia‐responsive miR‐141–3p was implicated in the pathogenesis of BC via mediating the HMGB1/HIF‐1α signaling pathway. Our preliminary findings revealed that the expression of miR‐141–3p was blocked by hypoxia in human BC. Moreover, the overexpression of miR‐141–3p could counteract hypoxia‐induced cell proliferation and migration through the suppression of the HMGB1/HIF‐1α axis.

## 2 MATERIALS AND METHODS

### 2.1 Cell culture

BC cell lines (MDA‐MB‐231 and MCF‐7) and human normal mammary epithelial cell line (MCF‐10A) were obtained from the American Type Culture Collection (ATCC, Manassas, VA, USA) and then cultured in the medium consisting of RPMI 1640 (Life Technologies, Carlsbad, CA, USA) supplemented with penicillin G (100 units/ml), streptomycin (100 mg/ml) and 10% fetal bovine serum (Life Technologies) with a 5% CO_2_ atmosphere at 37°C. For hypoxia stimulation, MDA‐MB‐231 and MCF‐7 cells were incubated in a hypoxia chamber with 1% oxygen for varying exposure times.

### 2.2 Analysis of cell viability using a 3‐(4,5‐dimethylthiazol‐2‐yl)‐2,5‐diphenyltetrazolium bromide (MTT) assay

Cell (2 × 10^5^ per well) viability was measured by use of an MTT Cell Proliferation/Viability Assay kit (R&D Systems, Minneapolis, MN, USA) in accordance with the manufacturer's instructions.

### 2.3 Cell transfection

Small interfering RNA was designed to silence HMGB1 expression. Si‐Con and si‐HMGB1 were synthesized by GenePharma (Shanghai, China). Si‐Con (empty plasmid) and si‐HMGB1 (5'‐GCTCAGACATTGTAGGATT‐3') were transfected into BC cells by Lipofectamine 2000 (Invitrogen, Carlsbad, CA, USA) for 48 hours at 37°C in accordance with the manufacturer's instructions.

### 2.4 Microarray assays

A miRNA microarray analysis was performed using Agilent Human miRNA (8 × 15K) V14.0 arrays of Ribobio (Guangzhou, China), as noted above.^18^ Concisely, probes (60‐mer) were synthesized using ink‐jet chemical in situ synthesis. A labeling and hybridization kit (Agilent Technologies, Santa Clara, CA, USA; 20 hours at 55°C) was used to detect miRs expression, then analyzed using Agilent Feature Extraction software, version 10.7.3.1 (Agilent Technologies). Differentially expressed miRs were selected out according to log_2_ (fold change) ≥ 1.0 or log_2_ (fold change) ≤ −1.0, *p* < 0.01 and false discovery rate ≤ 0.01. The hierarchical cluster analysis was performed using MeV, version 4.2.6.^19^

### 2.5 Quantitative reverse transcription‐polymerase chain reaction (qRT‐PCR)

The miRNeasy Mini Kit (Qiagen, Inc., Valencia, CA, USA) was used to extract total RNA. A TaqMan® RT kit and a TaqMan® MicroRNA assay (Applied Biosystems, Foster City, CA, USA) were used to detect miR‐141–3p expression levels with an Applied Biosystems 7,300 Real‐Time PCR System (Thermo Fisher Scientific, Inc., Waltham, MA, USA) in accordance with the manufacturer's instructions. The thermocycling conditions for the PCR were: 95°C for 10 minutes, 40 cycles of 95°C for 15 seconds and 60°C for 60 seconds. We adopted the 2^–ΔΔCt^ method to calculate miR‐141–3p expression levels as described previously,^20^ and used U6 as an internal control. The PCR primers were: forward, 5'‐GCTAACACTGTCTGGTAA‐3' and reverse, 5'‐CAGTGCGTGTCGTGGAGT‐3' for miR‐141–3p; forward, 5'‐CTCGCTTCGGCAGCACA‐3' and reverse, 5'‐ AACGCTTCACGAATTTGCGT‐3' for U6.

### 2.6 Migration and invasion assays

Migration of cells (2 × 10^4^) was analyzed with the transwell chamber (8 μm pore size; Corning Inc., Corning, NY, USA) without Matrigel matrix. Cells (2 × 10^4^) were seeded into the upper chamber pre‐coated with Matrigel matrix (BD Biosciences, Franklin Lakes, NJ, USA) for invasion analysis. After incubation for 24 hours, cells in the down chamber were stained with 0.1% crystal violet (Beyotime, Beijing, China) and photographed using an inverted fluorescence microscope (Leica Microsystems GmbH, Wetzlar, Germany). The transwell migration and invasion assay were carried out as described above.^21^

### 2.7 Immunohistochemical (IHC) staining and western blotting

Adjacent normal tissues (*n* = 74) and BC tissues (n = 74) were collected from the Shandong Provincial Hospital, Shandong University and Liaocheng People's Hospital. Informed consent forms had been obtained from the BC patients. Permission for the study was granted by the Ethics Committee of the Shandong Provincial Hospital, Shandong University and Liaocheng People's Hospital. Specimens were embedded in paraffin wax before being cut into 3‐μm slices for IHC staining. The protocols were performed as previously described.^22^ Image Pro‐Plus 6 software (Media Cybernetics, Inc., Rockville, MD, USA) was used to evaluate HMGB1‐positive staining. HMGB1 (catalog no. ab79823; dilution 1:100) was purchased from Abcam (Cambridge, UK). for IHC staining. Western blotting procedures were performed as described previously.^23^ The primary antibody for HMGB1 (catalog no. ab79823; dilution 1:1000) and HIF‐1α (catalog no. ab51608; dilution 1:1000) was obtained from Abcam. The secondary antibody conjugated with horseradish peroxidase (anti‐rabbit IgG‐HRP: sc‐2,357; incubation time: 2 h at room temperature) was purchased from Santa Cruz Biotechnology, Inc. (Dallas, TX, USA). Signals were analyzed using Quantity One, version 4.5 (Bio‐Rad Laboratories, Inc., Hercules, CA, USA). Anti‐β‐actin (catalog no. sc‐130065; dilution 1: 2000; Santa Cruz Biotechnology) was chosen as the control antibody.

### 2.8 Luciferase reporter assay

Wild‐type (WT) and mutant‐type (Mut) 3'‐UTR of HMGB1 were synthesized by Sangon (Shanghai, China) and inserted into the pmirGLO Dual‐Luciferase miRNA Target Expression Vector (Promega, Madidon, WI, USA). For the luciferase assay, MDA‐MB‐231 and MCF‐7 cells (1 × 10^5^) were seeded into 24 wells, and co‐transfected with luciferase reporter vectors containing WT or Mut 3'‐UTR (0.5 μg) of HMGB1 combined with miR‐Con or miR‐141–3p mimics (100 nmol/l) by Lipofectamine 2000 (Invitrogen) at 37°C for 48 hours. Luciferase activity was measured by a dual‐luciferase reporter assay kit (Beyotime Institute of Biotechnology, Beijing, China) in accordance with the manufacturer's instructions.

### 2.9 Nude‐mouse transplanted tumor model

Four‐week‐old male BALB/c nude mice were obtained from (*n* = 12) Beijing HFK Bio‐Technology. Co., Ltd (Beijing, China). Human BC MDA‐MB‐231 cells were stably transfected with miR‐Con (5'‐UCCCGGUUAUGAUUGUCUCGAG‐3') or miR‐141–3p mimics (5'‐UAACACUGUCUGGUAAAGAUGG‐3') by Lipofectamine 2000 (Invitrogen; Thermo Fisher Scientific, Inc.) for 48 hours at 37°C in accordance with the manufacturer's instructions. MDA‐MB‐231 cells (1 × 10^7^ cells per 0.1 mL) were injected subcutaneously into the same side armpit of each nude mouse anesthetized with 40 mg/kg sodium pentobarbital (Sigma‐Aldrich; Merck KGaA, Darmstadt, Germany). Nude mice were killed by inhalation of carbon dioxide. Tumor volumes and weight were measured at 5 weeks after cell implantation. The animal experiment was performed at the Shandong University Laboratory Animal Center (Jinan, China). The Ethics Committee of Shandong University (Jinan, China) approved the animal experiment (Approval number: 20190023).

### 2.10 Statistical analysis

Data are expressed as the mean ± SD. Statistical analysis was performed using Prism, version 7.0 (GraphPad Software, Inc., La Jolla, CA, USA). Chi‐squared tests were conducted to evaluate differences between clinical features and miR‐141–3p expression. Spearman's rank analysis was used to identify the correlation between miR‐141–3p and HMGB1 in BC tissues. Univariate and multivariate regression analysis were carried out to evaluate the correlation between miR‐141–3p expression and overall survival using a Cox proportional hazard model. Furthermore, we used Student’s *t* test to compare the differences between these two groups. Inter‐group comparisons were made using one‐way analysis of variance with Tukey's post‐hoc analysis. The Kaplan–Meier method, together with the log‐rank test, was used to estimate survival probabilities and compare survival between groups. *p* < 0.05 was considered statistically significant.

## 3 RESULTS

### 3.1 Hypoxia‐responsive miRs in BC MDA‐MB‐231 cells

**FIGURE 1:**
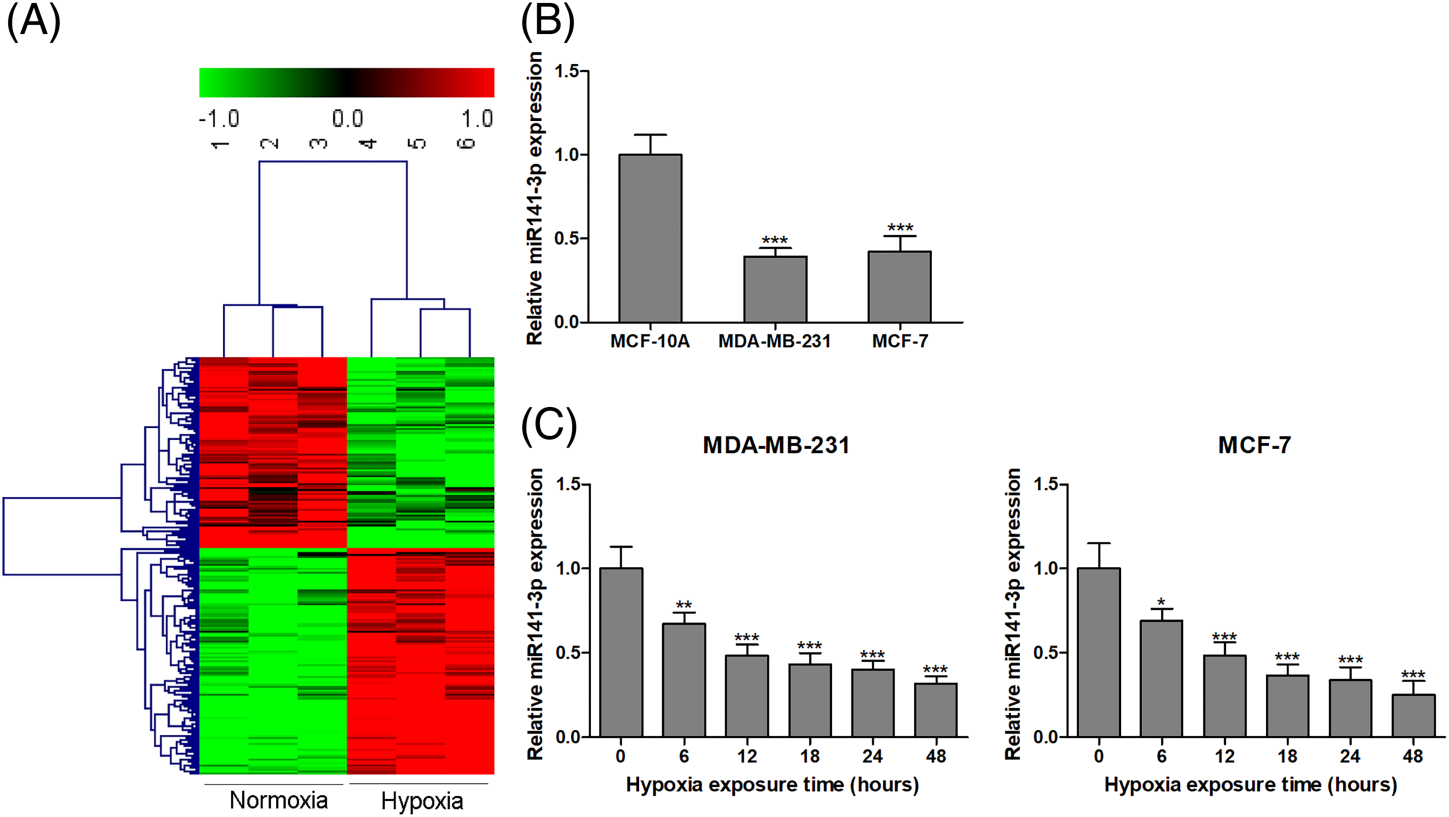
Hypoxia‐responsive miRs in BC MDA‐MB‐231 cells. Differentially expressed miRs in response to hypoxia condition were analyzed using miRs microarray assays (A). The expression of miR‐141–3p was detected in the two BC cell lines (MDA‐MB‐231 and MCF‐7) and human normal mammary epithelial cell MCF‐10A by qRT‐PCR (B). The abundance of miR‐141–3p was progressively gradually declined in MDA‐MB‐231 and MCF‐7 cells in a time‐dependent manner after exposure to hypoxia (C). ^*^ *p* < 0.05, ^**^ *p* < 0.01, ^***^ *p* < 0.001 compared to the corresponding control group. *n* = 3 in each group

To determine the role of hypoxia‐responsive miRs in the progression of BC, differentially expressed miRs in response to hypoxia conditions were analyzed using miR microarray assays. Hypoxia exposure led to 212 miRs that exhibited significant differential expression, including 97 down‐regulated miRs and 115 up‐regulated miRs, according to a log_2_ (fold change) ≥ 1.0 or log_2_ (fold change) ≤ −1.0, *p* < 0.01 and false discovery rate ≤ 0.01 (Figure 1A). miR‐141–3p [log_2_ (fold change) = −4.69] as the most valuable nominated miR was focused in our further studies. Moreover, we confirmed the expression of miR‐141–3p was significantly decreased in the two BC cell lines compared to the MCF‐10A human normal mammary epithelial cell line (Figure 1B). We also found that the abundance of miR‐141–3p was progressively gradually declined in MDA‐MB‐231 and MCF‐7 human BC cells in a time‐dependent manner after exposure to hypoxia (Figure 1C).

### 3.2 miR‐141–3p is an independent prognostic factor predicting overall survival in BC patients

**FIGURE 2:**
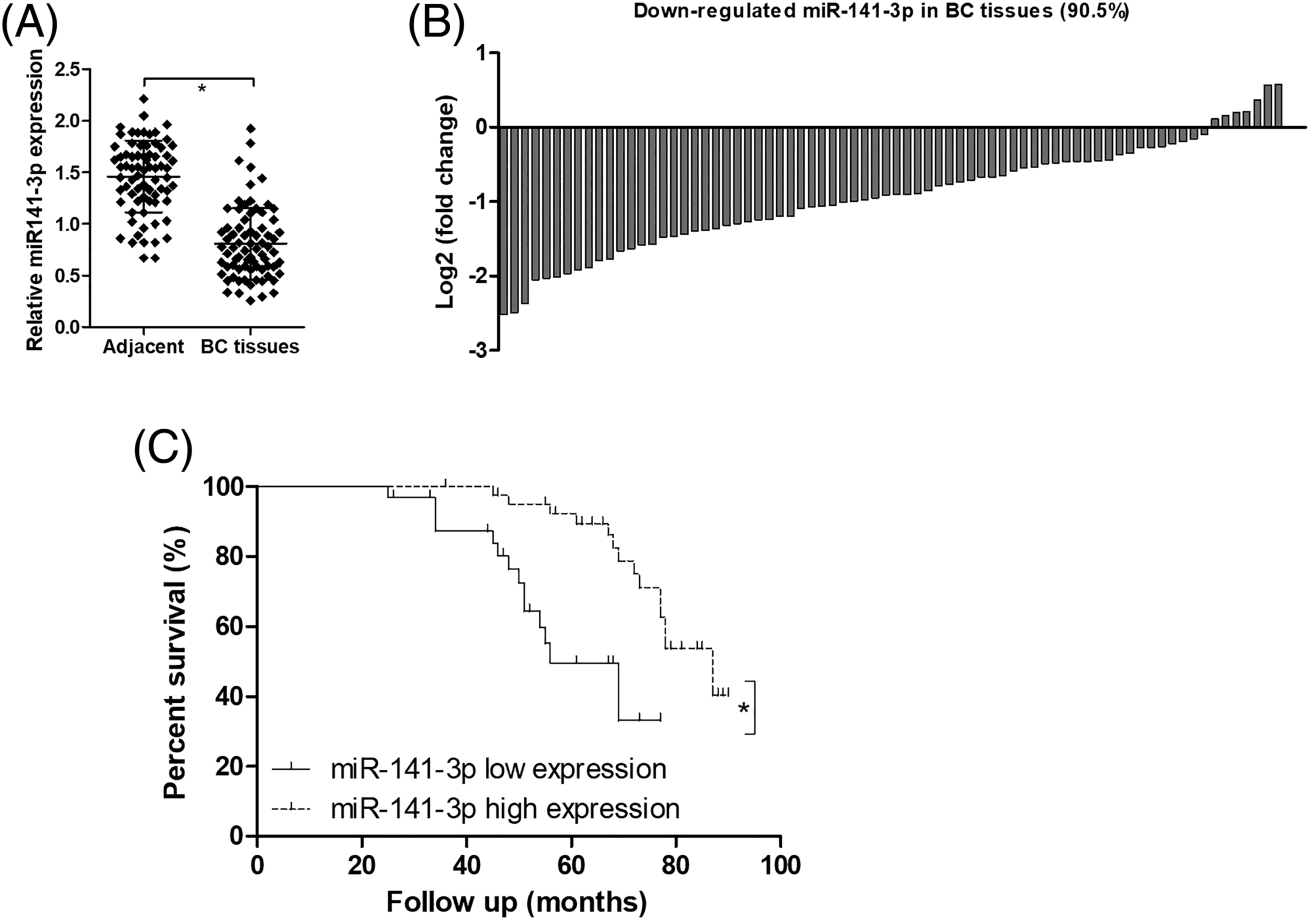
miR‐141–3p is an independent prognostic factor for predicting the overall survival of BC patients. The expression of miR‐141–3p was detected in 74 pairs of BC tissues and adjacent non‐tumor tissues using qRT‐PCR assays (A) (*n* = 74), and miR‐141–3p was down‐regulated in 67 of 74 (90.5%) BC specimens (B) (*n* = 74). We defined that miR‐141–3p low expression was a log_2_ (fold change) ≤ −1 (*n* = 33), and high expression of miR‐141–3p was a log_2_ (fold change) > −1 (*n* = 41). Patients with low expression of miR‐141–3p showed a shorter overall survival than those of patients with miR‐141–3p high expression (C). ^*^ *p* < 0.05

First, we detected the expression of miR‐141–3p in 74 pairs of BC tissues and adjacent normal tissues. qRT‐PCR assays demonstrated that miR‐141–3p was significantly decreased in BC tissues compared to adjacent normal tissues, and miR‐141–3p was down‐regulated in 67 of 74 (90.5%) BC specimens (Figure 2A and 2B). Because we defined that miR‐141–3p low expression was a log_2_ (fold change) ≤ −1, the results demonstrated that miR‐141–3p showed low expression in 33 BC specimens and high expression in 41 BC specimens. Intriguingly, patients with low expression of miR‐141–3p showed a shorter overall survival than patients with miR‐141–3p high expression (Figure 2C). Moreover, miR‐141–3p low expression was associated with tumor sizes, TNM stages and lymph nodes metastasis in BC patients (Table 1). Univariate and multivariate regression analysis indicated that tumor size, TNM stages, lymph nodes metastasis and miR‐141–3p could also serve as independent prognostic factors predicting the overall survival of BC patients (Table 2).

**TABLE 1:** Correlation between clinicopathological parameters and miR‐141–3p expression levels in breast cancer patients

Variable	n	miR‐141–3p expression		*p* value
		Low (*n* = 33)	High (*n* = 41)	
Age (years)				0.514
< 50	35	17	18	
≥ 50	39	16	23	
Tumor size (cm)				0.007
< 2	42	13	29	
≥ 2	32	20	12	
TNM stages				< 0.001
I‐II	43	11	32	
III‐IV	31	22	9	
Lymph nodes metastasis				0.001
Negative (N)	47	14	33	
Positive (P)	27	19	8	

**TABLE 2:** Univariate and multivariate regression analysis of breast cancer patients for overall survival

Variables	Univariate		Multivariate	
	HR (95% CI)	*p* value	HR (95% CI)	*p* value
Age (≥ 50 versus < 50)	0.95 (0.51–1.92)	0.738		
Tumor size (≥ 2 versus < 2)	2.67 (1.54–7.01)	0.009	2.22 (1.23–6.35)	0.014
TNM stages (III–IV versus I–II)	3.36 (1.73–10.57)	< 0.001	2.61 (1.43–7.21)	0.006
Lymph nodes metastasis (P versus N)	3.24 (1.69–9.93)	< 0.001	2.43 (1.37–6.91)	0.01
miR‐141–3p (low versus high)	2.98 (1.59–8.71)	0.002	2.29 (1.33–6.79)	0.011

HR, hazard ratio; CI, confidence interval.

### 3.3 Overexpression of miR‐141–3p attenuates hypoxia‐induced cell migration

**FIGURE 3:**
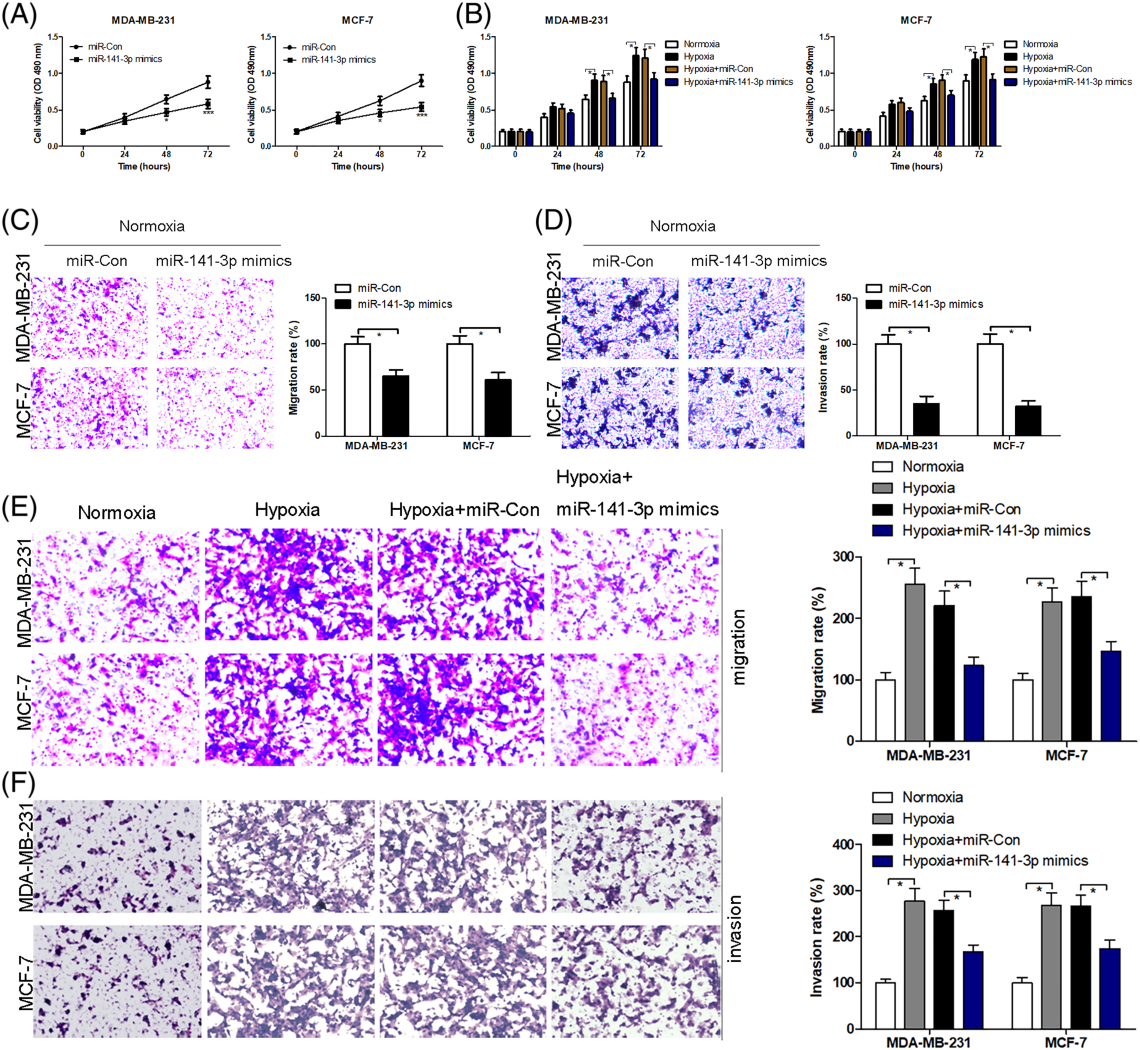
Overexpression of miR‐141–3p attenuates hypoxia‐induced cell migration. After transfection with miR‐141–3p mimics into MDA‐MB‐231 and MCF‐7 cells under conditions of normoxia (A) or hypoxia (B), the MTT assay was used to evaluate cell proliferation. After transfection with miR‐141–3p mimics into MDA‐MB‐231 and MCF‐7 cells under conditions of normoxia for 24 hours, cell migration (C) and invasion (D) was evaluated by transwell assays. After transfection with miR‐con or miR‐141–3p mimics into MDA‐MB‐231 and MCF‐7 exposure to hypoxia condition for 24 hours, cell migration (E) and invasion (F) was evaluated by transwell assays. ^*^ *p* < 0.05. *n* = 3 in each group

Previous studies highlight that hypoxia is a key stimulant of BC cell proliferation, migration and invasion.^16^, ^24^ After transfection with miR‐141–3p mimics into MDA‐MB‐231 and MCF‐7 cells under normoxic or hypoxic conditions, the MTT assay was used to evaluate cell proliferation. In normoxia conditions, transfection with miR‐141–3p mimics into MDA‐MB‐231 and MCF‐7 cells resulted in significant inhibition of cell proliferation compared to that of in the miR‐Con group (Figure 3A). When MDA‐MB‐231 and MCF‐7 cells were under hypoxia conditions, cell proliferation was dramatically enhanced. However, transfection with miR‐141–3p mimics significantly attenuated hypoxia‐evoked cell proliferation (Figure 3B). As shown in Figure 3C and 3D, overexpression of miR‐141–3p effectively suppressed the migration and invasion of MDA‐MB‐231 and MCF‐7 cells under normoxia conditions compared to that in the miR‐Con group. To investigate the role of miR‐141–3p in hypoxia‐induced cell migration, transwell assays were utilized to analyze BC cell migration and invasion. A significant increase in cell migration and invasion was observed in hypoxia‐stimulated MDA‐MB‐231 and MCF‐7 cells compared to those under normoxia conditions (Figure 3E and 3F). However, transfection with miR‐141–3p mimics into MDA‐MB‐231 and MCF‐7 cells notably reduced the abilities of hypoxia‐induced cell migration and invasion (Figure 3E and 3F).

### 3.4 Overexpression of miR‐141–3p inhibits the hypoxia‐activated HMGB1/HIF‐1α signaling pathway

**FIGURE 4:**
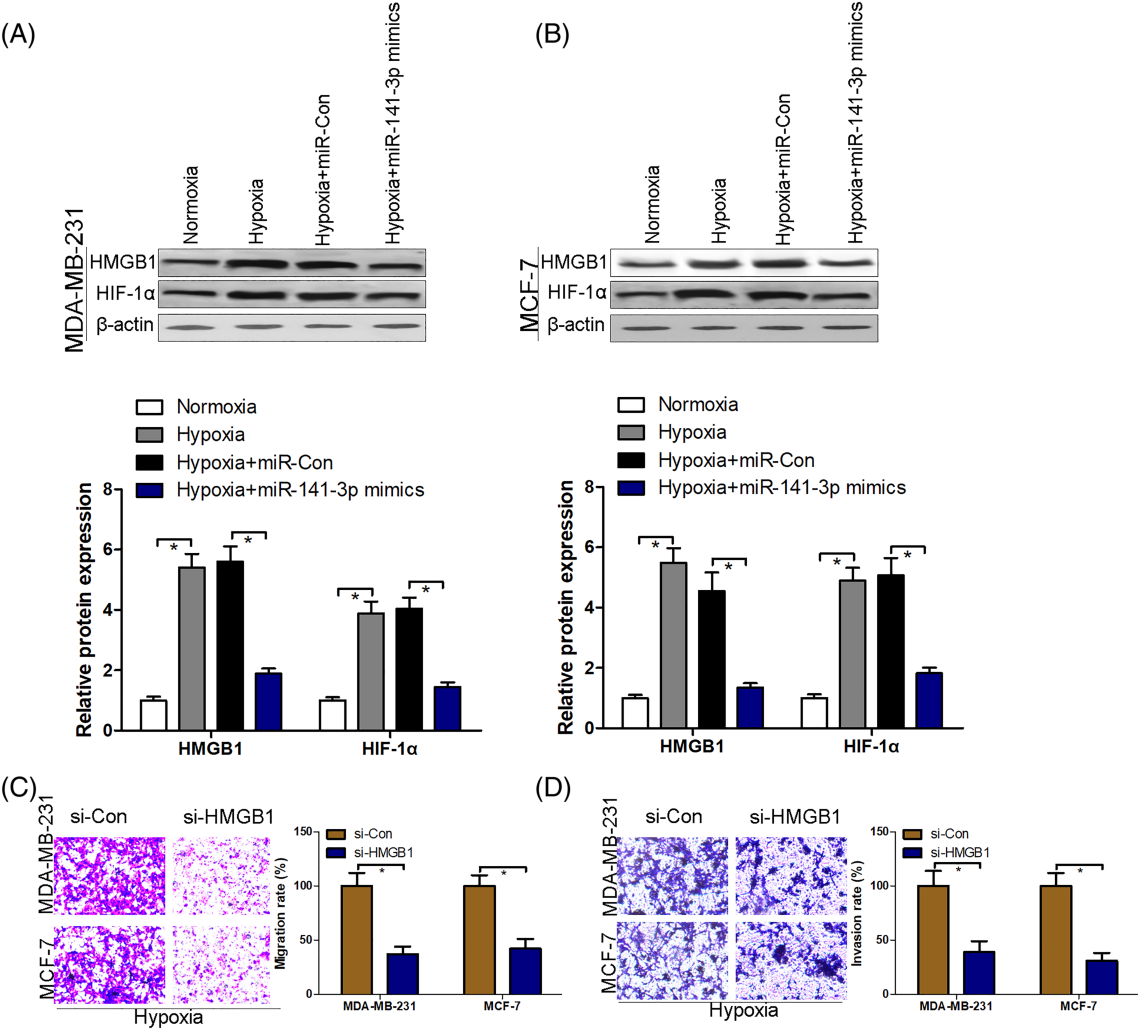
Overexpression of miR‐141–3p inhibits the hypoxia‐activated HMGB1/HIF‐1α signaling pathway. After transfection with miR‐con or miR‐141–3p mimics into MDA‐MB‐231 and MCF‐7 exposure to hypoxia condition for 48 hours, protein expression of HMGB1 and HIF‐1α was measured using western blotting (A and B). After transfection with si‐con or si‐HMGB1 into MDA‐MB‐231 and MCF‐7 cells under conditions of hypoxia for 24 hours, cell migration (C) and invasion (D) were evaluated by transwell assays. ^*^ *p* < 0.05. *n* = 3 in each group

HMGB1 has been corroborated as an oncogene with respect to the initiation and progression of various cancers.^10^, ^25^ HMGB1‐modulated HIF‐1α signaling pathway can promote angiogenesis and tumor migration.^10^, ^25^ The present study aimed to investigate whether miR‐141–3p could mediate HMGB1/HIF‐1α signaling pathway in hypoxia‐exposed BC cells. Our findings showed that protein expression of HMGB1 and HIF‐1α was significantly increased in hypoxia‐exposed BC cells compared to those under normoxia conditions. However, hypoxia‐induced up‐regulation of HMGB1 and HIF‐1α protein expression was partially neutralized after the transfection of miR‐141–3p mimics into MDA‐MB‐231 and MCF‐7 cells (Figure 4A and 4B). After transfection with si‐Con or si‐HMGB1 (50 nmol/l) into MDA‐MB‐231 and MCF‐7 cells for 48 h, cell migration and invasion were analyzed using transwell assays. Under hypoxia conditions, the silencing of HMGB1 suppressed the migration and invasion of MDA‐MB‐231 and MCF‐7 cells (Figure 4C and 4D).

### 3.5 HMGB1 is a direct target of miR‐141–3p

**FIGURE 5:**
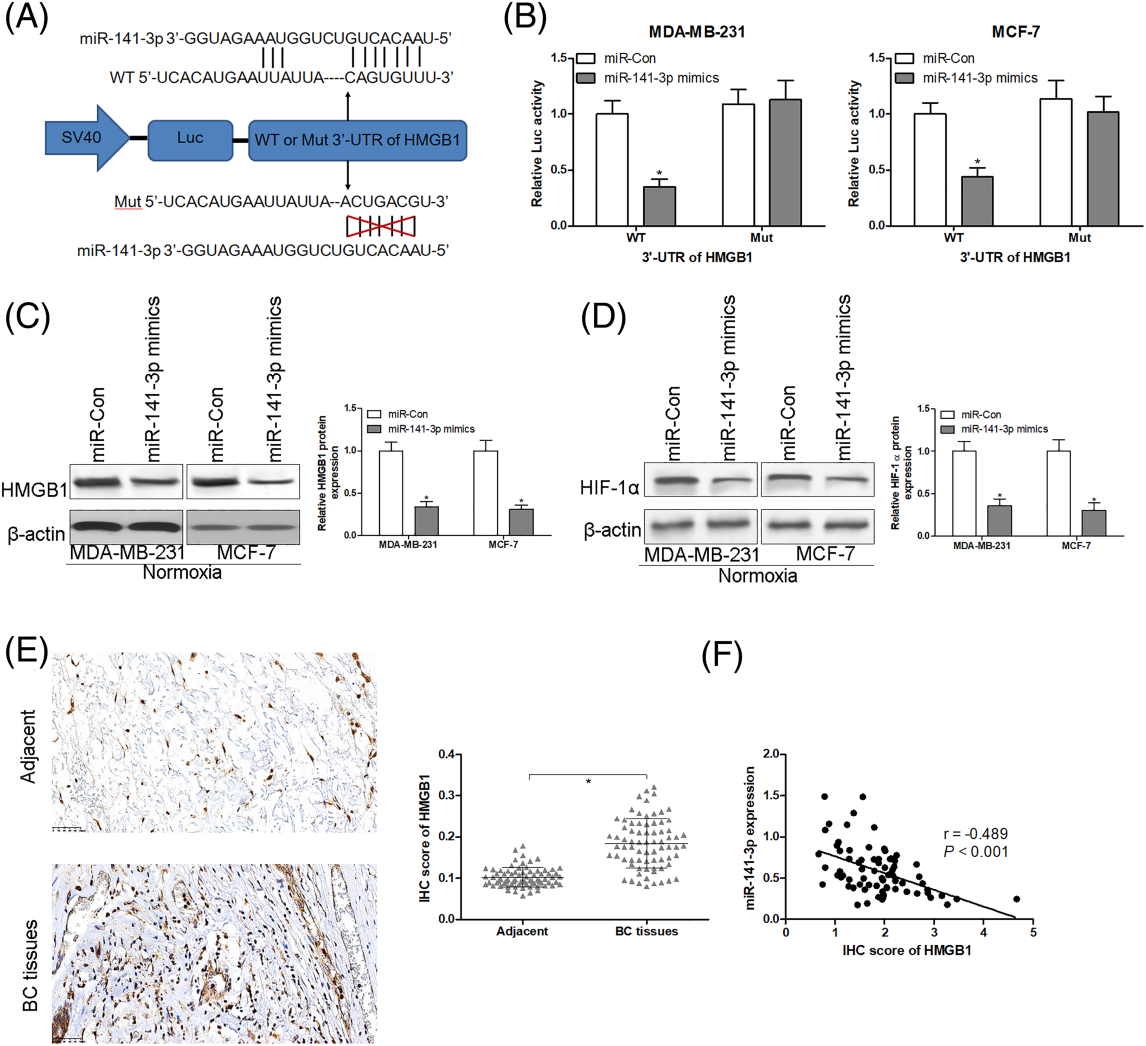
HMGB1 is a direct target of miR‐141–3p. On‐line bioinformatics algorithms (Targetscan; www.targetscan.org) revealed that a conserved sequence in the 3'‐UTR of HMGB1 could be bonded with miR‐141–3p (A). A direct interaction between miR‐141–3p and the 3'‐UTR of HMGB1 was analyzed using a luciferase reporter assay (B) (*n* = 3 in each group.). After transfection with miR‐con or miR‐141–3p mimics into MDA‐MB‐231 and MCF‐7 for 48 hours, protein expression of HMGB1 (C) and HIF‐1α (D) was measured using western blotting (*n* = 3 in each group.). Protein expression of HMGB1 was detected using IHC staining in 74 pairs of adjacent non‐tumor tissues and BC tissues (E) (*n* = 74 in each group.). Pearson's correlation analysis showed that the expression of HMGB1 was significantly and inversely correlated with miR‐141–3p in BC tissues (F) (*n* = 74 in each group.). ^*^ *p* < 0.05 compared to the corresponding control group

According to the above conclusions, our results indicate that miR‐141–3p can regulate HMGB1 protein expression, yet the post‐transcriptional repression role of miR‐141–3p on HMGB1 protein expression remains unclear. Bioinformatics algorithms revealed that a conserved sequence in the 3'‐UTR of HMGB1 could bond with miR‐141–3p, as shown in Figure 5A. In addition, direct interaction between miR‐141–3p and the 3'‐UTR of HMGB1 was analyzed using a luciferase reporter assay. After transfection with miR‐141–3p mimics, luciferase activity was significantly reduced in MDA‐MB‐231 and MCF‐7 cells containing WT 3'‐UTR of HMGB1 compared to those transfected with miR‐Con. However, the luciferase activity had no notable change in MDA‐MB‐231 and MCF‐7 cells containing Mut 3'‐UTR of HMGB1 after transfection with miR‐Con or miR‐141–3p mimics (Figure 5B). The findings indicated that HMGB1 is a direct target of miR‐141–3p. Furthermore, our results showed a significant decrease in HMGB1 protein expression in MDA‐MB‐231 and MCF‐7 cells after transfection with miR‐141–3p mimics (Figure 5C), suggesting that miR‐141–3p could inhibit HMGB1 protein expression by post‐transcriptional repression. We have discovered that miR‐141–3p mimics transfection significantly reduced protein expression of HIF‐1α compared to the control group under conditions of normoxia (Figure 5D). Moreover, IHC staining indicated a significant increase in HMGB1 protein in BC tissues (Figure 5E), and a significant inverse correlation between HMGB1 protein level and miR‐141–3p expression was observed in 74 BC tissues (Figure 5F).

### 3.6 miR‐141–3p inhibits the HMGB1/HIF‐1α signaling pathway *in vivo*

**FIGURE 6:**
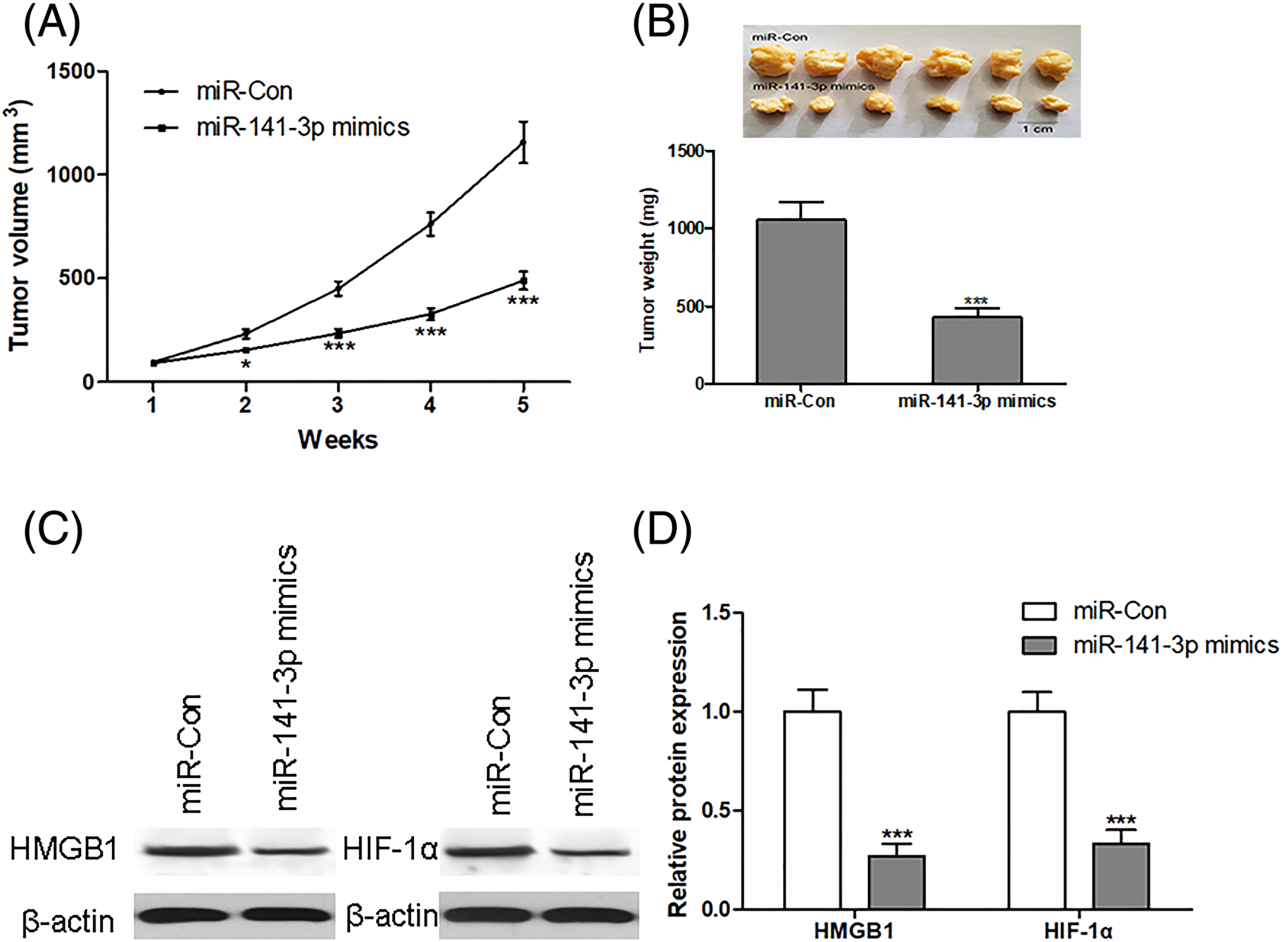
miR‐141–3p inhibits the HMGB1/HIF‐1α signaling pathway *in vivo*. BC MDA‐MB‐231 cells (1 × 10^7^ cells per 0.1 mL) were established to steadily express miR‐141–3p. MDA‐MB‐231 cells were implanted subcutaneously into 4‐week‐old BALB/c nude mice, and tumor volume and weight were evaluated after 5 weeks with MDA‐MB‐231 cell implantation (A and B). Protein expression of HMGB1 and HIF‐1α in solid tumors was measured using western blotting (C and D). ^*^ *p* < 0.05, ^***^ *p* < 0.001 compared to the corresponding control group. *n* = 6 in each group

The antineoplastic role of miR‐141–3p in the nude mouse transplanted tumor model was investigated. BC MDA‐MB‐231 cells (1 × 10^7^ cells per 0.1 ml) were established to steadily express miR‐141–3p. MDA‐MB‐231 cells were implanted into 4‐week‐old BALB/c nude mice subcutaneously, and tumor growth was evaluated 5 weeks after MDA‐MB‐231 cell implantation. The tumor volume and weight were significantly inhibited in miR‐141–3p overexpressed mice compared to the control group (Figure 6A and 6B). We also found that overexpression of miR‐141–3p could inhibit protein expression of HMGB1 and HIF‐1α solid tumors (Figure 6C and 6D).

## 4 DISCUSSION

As a consequence of solid tumor growth, hypoxia has been frequently reported in various malignant tumors.^5^, ^16^, ^24^ Meanwhile, the hypoxic condition is a key factor with respect to promoting tumor migration, invasion, metastasis and chemotherapy‐resistance, leading to a poorer prognosis.^5^, ^16^, ^24^ Indeed, hypoxia can stabilize HIF1α at the post‐translational level to stimulate malignant processes, including angiogenesis, migration and invasion, via up‐regulating the expression of hypoxically regulated genes, such as the vascular endothelial growth factor and matrix metalloproteinases.^24^, ^26^ Intriguingly, some specific miRs can be regulated by hypoxia in the initiation and progression of cancers.^5^ For example, overexpression of miR‐210 is induced by hypoxia and exhibits a significant inverse correlation with disease‐free and overall survival in BC patients.^5^, ^16^, ^27^ As hypoxia‐responsive miRs, miR‐25 and miR‐93 are implicated in hypoxia‐induced immunosuppression in BC.^27^ Hypoxia‐induced miR‐153 contributes to angiogenesis in BC.^16^ These findings suggest that hypoxia‐related miRs play an important role in cancer progression.

In the present study, miR‐141–3p was observed as a hypoxia‐responsive miR in BC. miR‐141–3p was down‐regulated in BC specimens and could serve as an independent prognostic factor predicting overall survival in BC patients. In addition, overexpression of miR‐141–3p could inhibit hypoxia‐induced cell migration and impede human BC MDA‐MB‐231 cell growth *in vivo.* Mechanistically, hypoxia‐related HMGB1/HIF‐1α signaling pathway might be a potential target of miR‐141–3p to prevent the progression of BC. Choi *et al*.^28^ highlighted that miR‐141/200 family members, miR‐200a/b/c, were found to effectively repress growth and significantly decrease migration and invasion of MDA‐MB‐231 cell *in vitro.* These findings demonstrate that miR‐141/200 family members perform multi‐faceted roles in BC.

miR‐141–3p as a tumor suppressor has been reported in multiple cancer types, including colorectal cancer, lung cancer and BC.^29^, ^30^, ^31^, ^32^ In BC, overexpression of miR‐141–3p could enhance the sensitivity of BC cells to trastuzumab via post‐transcriptional repression of cyclin‐dependent kinase 8.^30^ Furthermore, the overexpression of miR‐141–3p could suppress epithelial‐mesenchymal transition in BC cells by targeting zinc‐finger E‐box binding homeobox 1 (ZEB1) and ZEB2.^29^ In the present study, we extended the function of miR‐141–3p, which could blunt BC cell migration under hypoxic conditions.

Prior studies have revealed that HMGB1 is persistently associated with hypoxia to promote tumor metastasis.^33^ In the process of HCC, hypoxia exposure gives rise to HMGB1 expression to mediate mitochondrial biogenesis and stimulate macrophage‐derived interleukin‐6, which promotes tumor growth and enhances the invasiveness and metastasis of hepatoma cells.^33^, ^34^ HMGB1 is notably increased in the serum of patients with metastatic melanoma and is released by melanoma cells under hypoxic conditions, promoting melanoma growth and metastasis.^35^ However, the role of HMGB1 in the hypoxia‐induced migration of BC cells is still unclear. We established a tumor microenvironment of BC cells *in vitro* and found that hypoxia exposure promoted BC cell migration and activated HMGB1/HIF‐1α signaling pathway. In a further exploration of the molecular mechanisms underlying the hypoxia‐induced aggressive tumor phenotype of BC, we found that, as a potential tumor suppressor, miR‐141–3p counteracted hypoxia‐induced migration and suppressed the hypoxia‐activated HMGB1/HIF‐1α pathway. Bioinformatics analysis and *in vitro* experimental measurement revealed that miR‐141–3p could bind with the 3'‐UTR of HMGB1, and overexpression of miR‐141–3p led to a significant decrease of HMGB1 protein expression in MDA‐MB‐231 and MCF‐7 cells. Not surprisingly, Spearman's rank analysis showed a significant and negative correlation between miR‐141–3p and HMGB1 protein expression in BC tissues. These findings directly or indirectly validate HMGB1 as a target of miR‐141–3p that can repress HMGB1 protein expression in a post‐transcriptional manner.

In conclusion, the present study has study highlighted that miR‐141–3p could act as an independent prognostic factor for BC and also revealed that overexpression of miR‐141–3p plays an antineoplastic activity by suppressing hypoxia‐induced cell migration *in vitro* and solid tumor growth *in vivo* via mediating the HMGB1/HIF‐1α pathway. Our findings provide a new mechanism for understanding hypoxia‐related BC progression and corroborate miR‐141–3p as a potential therapeutic target for the treatment of BC.

## AUTHOR CONTRIBUTIONS

SS and XT are guarantors for the integrity of the entire study. SS, XT and ZW were responsible for conceiving the study. SS and XT were responsible for the study design. ZW was responsible for the definition of intellectual content. JM was responsible for researching the literature. SS and PX were responsible for clinical studies. SS, JM, PX and ZW were responsible for experimental studies. JM and PX were responsible for data acquisition. JM and SS were responsible for data analysis. PX was responsible for statistical analysis. SS, JM, PX and ZW were responsible for manuscript preparation. SS, ZW and XT were responsible for editing the manuscript. SS, JM, PX, ZW and XT were responsible for reviewing the manuscript.

## CONFLICT OF INTEREST STATEMENT

The authors declare that they have no conflicts of interest.

The datasets used and/or analyzed during the present study are available from the corresponding author upon reasonable request.

## References

[1] Bray F , Ferlay J , Soerjomataram I , Siegel RL , Torre LA , Jemal A . Global cancer statistics 2018: GLOBOCAN estimates of incidence and mortality worldwide for 36 cancers in 185 countries. CA Cancer J Clin. 2018;68:394‐424. 30207593 10.3322/caac.21492

[2] Feng RM , Zong YN , Cao SM , Xu RH . Current cancer situation in China: good or bad news from the 2018 global cancer statistics? Canc Comm (London, England). 2019;39:22. 10.1186/s40880-019-0368-6 PMC6487510 31030667

[3] Chen W , Zheng R , Baade PD , et al. Cancer statistics in China, 2015. CA Cancer J Clin. 2016;66:115‐132. 26808342 10.3322/caac.21338

[4] Daimiel I . Insights into hypoxia: non‐invasive assessment through imaging modalities and its application in breast cancer. J Breast Cancer. 2019;22:155‐171. 31281720 10.4048/jbc.2019.22.e26 PMC6597408

[5] Camps C , Buffa FM , Colella S , et al. Hsa‐miR‐210 is induced by hypoxia and is an independent prognostic factor in breast cancer. Clin Cancer Res. 2008;14:1340‐1348. 18316553 10.1158/1078-0432.CCR-07-1755

[6] Cox TR , Rumney RMH , Schoof EM , et al. The hypoxic cancer secretome induces pre‐metastatic bone lesions through lysyl oxidase. Nature. 2015;522:106‐110. 26017313 10.1038/nature14492 PMC4961239

[7] Facciabene A , Peng X , Hagemann IS , et al. Tumour hypoxia promotes tolerance and angiogenesis via CCL28 and T (reg) cells. Nature. 2011;475:226‐230. 21753853 10.1038/nature10169

[8] Nardinocchi L , Puca R , Sacchi A , D'Orazi G . Inhibition of HIF‐1alpha activity by homeodomain‐interacting protein kinase‐2 correlates with sensitization of chemoresistant cells to undergo apoptosis. Mol Cancer. 2009;8:1. 19128456 10.1186/1476-4598-8-1 PMC2628864

[9] Park SY , Lee SW , Kim HY , Lee WS , Hong KW , Kim CD . HMGB1 induces angiogenesis in rheumatoid arthritis via HIF‐1alpha activation. Eur J Immunol. 2015;45:1216‐1227. 25545169 10.1002/eji.201444908

[10] He H , Wang X , Chen J , Sun L , Sun H , Xie K . High‐mobility group box 1 (HMGB1) promotes angiogenesis and tumor migration by regulating hypoxia‐inducible factor 1 (HIF‐1alpha) expression via the phosphatidylinositol 3‐kinase (PI3K)/AKT signaling pathway in breast cancer cells. Med Sci Mon: Int Med J Exp Clin Res. 2019;25:2352‐2360. 10.12659/MSM.915690 PMC6454982 30930461

[11] Ueda T , Volinia S , Okumura H , et al. Relation between microRNA expression and progression and prognosis of gastric cancer: a microRNA expression analysis. Lancet Oncol. 2010;11:136‐146. 20022810 10.1016/S1470-2045(09)70343-2 PMC4299826

[12] Zhihua Y , Yulin T , Yibo W , et al. Hypoxia decreases macrophage glycolysis and M1 percentage by targeting microRNA‐30c and mTOR in human gastric cancer. Cancer Sci. 2019;110:2368‐2377. 31222863 10.1111/cas.14110 PMC6676118

[13] Wang W , Liu M , Guan Y , Wu Q . Hypoxia‐responsive Mir‐301a and Mir‐301b promote Radioresistance of prostate cancer cells via downregulating NDRG2. Med Sci Mon: Int Med J Exp Clin Res. 2016;22:2126‐2132. 10.12659/MSM.896832 PMC4920099 27327120

[14] Che J , Wang W , Huang Y , et al. miR‐20a inhibits hypoxia‐induced autophagy by targeting ATG5/FIP200 in colorectal cancer. Mol Carcinog. 2019;58:1234‐1247. 30883936 10.1002/mc.23006

[15] Costa V , Lo Dico A , Rizzo A , et al. MiR‐675‐5p supports hypoxia induced epithelial to mesenchymal transition in colon cancer cells. Oncotarget. 2017;8:24292‐24302. 28061476 10.18632/oncotarget.14464 PMC5421847

[16] Liang H , Xiao J , Zhou Z , et al. Hypoxia induces miR‐153 through the IRE1alpha‐XBP1 pathway to fine tune the HIF1alpha/VEGFA axis in breast cancer angiogenesis. Oncogene. 2018;37:1961‐1975. 29367761 10.1038/s41388-017-0089-8 PMC5895606

[17] Tang T , Yang Z , Zhu Q , et al. Up‐regulation of miR‐210 induced by a hypoxic microenvironment promotes breast cancer stem cells metastasis, proliferation, and self‐renewal by targeting E‐cadherin. FASEB J. 2018;32:6965‐6981. 10.1096/fj.201801013R 30188754

[18] Sun Y , Mei H , Xu C , Tang H , Wei W . Circulating microRNA‐339‐5p and ‐21 in plasma as an early detection predictors of lung adenocarcinoma. Pathol Res Pract. 2018;214:119‐125. 29103767 10.1016/j.prp.2017.10.011

[19] Bare JC , Shannon PT , Schmid AK , Baliga NS . The Firegoose: two‐way integration of diverse data from different bioinformatics web resources with desktop applications. BMC Bioinformatics. 2007;8(1):456 10.1186/1471-2105-8-456 18021453 PMC2211326

[20] Livak KJ , Schmittgen TD . Analysis of relative gene expression data using real‐time quantitative PCR and the 2(−Delta Delta C(T)) method. Methods (San Diego, Calif). 2001;25:402‐408. 10.1006/meth.2001.1262 11846609

[21] Li J , Guo Y , Duan L , et al. AKR1B10 promotes breast cancer cell migration and invasion via activation of ERK signaling. Oncotarget. 2017;8:33694‐33703. 28402270 10.18632/oncotarget.16624 PMC5464903

[22] Gong J , Wang ZX , Liu ZY . miRNA1271 inhibits cell proliferation in neuroglioma by targeting fibronectin 1. Mol Med Rep. 2017;16:143‐150. 28535003 10.3892/mmr.2017.6610 PMC5482146

[23] Yu FY , Xie CQ , Sun JT , Peng W , Huang XW . Overexpressed miR‐145 inhibits osteoclastogenesis in RANKL‐induced bone marrow‐derived macrophages and ovariectomized mice by regulation of Smad3. Life Sci. 2018;202:11‐20. 29577879 10.1016/j.lfs.2018.03.042

[24] Ren S , Liu J , Feng Y , et al. Knockdown of circDENND4C inhibits glycolysis, migration and invasion by up‐regulating miR‐200b/c in breast cancer under hypoxia. J Exp Clin Cancer Res: CR. 2019;38:388. 31488193 10.1186/s13046-019-1398-2 PMC6727545

[25] Chen R , Zhu S , Fan XG , et al. High mobility group protein B1 controls liver cancer initiation through yes‐associated protein ‐dependent aerobic glycolysis. Hepatology (Baltimore, md). 2018;67:1823‐1841. 10.1002/hep.29663 PMC5906197 29149457

[26] Sun L , Lin P , Qin Z , Liu Y , Deng LL , Lu C . Hypoxia promotes HO‐8910PM ovarian cancer cell invasion via snail‐mediated MT1‐MMP upregulation. Exp Biol Med (Maywood). 2015;240:1434‐1445. 25681470 10.1177/1535370215570205 PMC4935300

[27] Wu MZ , Cheng WC , Chen SF , et al. miR‐25/93 mediates hypoxia‐induced immunosuppression by repressing cGAS. Nat Cell Biol. 2017;19:1286‐1296. 28920955 10.1038/ncb3615 PMC5658024

[28] Choi SK , Kim HS , Jin T , Hwang EH , Jung M , Moon WK . Overexpression of the miR‐141/200c cluster promotes the migratory and invasive ability of triple‐negative breast cancer cells through the activation of the FAK and PI3K/AKT signaling pathways by secreting VEGF‐A. BMC Cancer. 2016;16:570. 27484639 10.1186/s12885-016-2620-7 PMC4969651

[29] Zhang Y , Li J , Jia S , Wang Y , Kang Y , Zhang W . Down‐regulation of lncRNA‐ATB inhibits epithelial‐mesenchymal transition of breast cancer cells by increasing miR‐141‐3p expression. Biochem Cell Biol. 2019;97:193‐200. 30352165 10.1139/bcb-2018-0168

[30] Song W , Wu S , Wu Q , et al. The microRNA‐141‐3p/CDK8 pathway regulates the chemosensitivity of breast cancer cells to trastuzumab. J Cell Biochem. 2019;120:14095‐14106. 31087707 10.1002/jcb.28685

[31] Liang Z , Li X , Liu S , Li C , Wang X , Xing J . MiR‐141‐3p inhibits cell proliferation, migration and invasion by targeting TRAF5 in colorectal cancer. Biochem Biophys Res Commun. 2019;514:699‐705. 31078266 10.1016/j.bbrc.2019.05.002

[32] Li W , Cui Y , Wang D , Wang Y , Wang L . MiR‐141‐3p functions as a tumor suppressor through directly targeting ZFR in non‐small cell lung cancer. Biochem Biophys Res Commun. 2019;509:647‐656. 30611568 10.1016/j.bbrc.2018.12.089

[33] Jiang J , Wang GZ , Wang Y , Huang HZ , Li WT , Qu XD . Hypoxia‐induced HMGB1 expression of HCC promotes tumor invasiveness and metastasis via regulating macrophage‐derived IL‐6. Exp Cell Res. 2018;367:81‐88. 29571949 10.1016/j.yexcr.2018.03.025

[34] Tohme S , Yazdani HO , Liu Y , et al. Hypoxia mediates mitochondrial biogenesis in hepatocellular carcinoma to promote tumor growth through HMGB1 and TLR9 interaction. Hepatology (Baltimore, md). 2017;66:182‐197. 10.1002/hep.29184 PMC5481489 28370295

[35] Huber R , Meier B , Otsuka A , et al. Tumour hypoxia promotes melanoma growth and metastasis via high mobility group Box‐1 and M2‐like macrophages. Sci Rep. 2016;6:29914. 27426915 10.1038/srep29914 PMC4947927

